# Sperry versus Hebb: Topographic mapping in Isl2/EphA3 mutant mice

**DOI:** 10.1186/1471-2202-11-155

**Published:** 2010-12-29

**Authors:** Dmitry Tsigankov, Alexei A Koulakov

**Affiliations:** 1 Cold Spring Harbor Laboratory, Cold Spring Harbor, NY 11724, USA; 2 Max-Planck Institute for Dynamics and Self-Organization, Gottingen, 37073, Germany; 3 Bernstein Center for Computational Neuroscience, Gottingen, 37073, Germany

## Abstract

**Background:**

In wild-type mice, axons of retinal ganglion cells establish topographically precise projection to the superior colliculus of the midbrain. This means that axons of neighboring retinal ganglion cells project to the proximal locations in the target. The precision of topographic projection is a result of combined effects of molecular labels, such as Eph receptors and ephrins, and correlated neural activity. In the Isl2/EphA3 mutant mice the expression levels of molecular labels are changed. As a result the topographic projection is rewired so that the neighborhood relationships between retinal cell axons are disrupted.

**Results:**

Here we study the computational model for retinocollicular connectivity formation that combines the effects of molecular labels and correlated neural activity. We argue that the effects of correlated activity presenting themselves in the form of Hebbian learning rules can facilitate the restoration of the topographic connectivity even when the molecular labels carry conflicting instructions. This occurs because the correlations in electric activity carry information about retinal cells' origin that is independent on molecular labels. We argue therefore that partial restoration of the topographic property of the retinocollicular projection observed in Isl2/EphA3 heterozygous knockin mice may be explained by the effects of correlated neural activity. We address the maps observed in Isl2/EphA3 knockin/EphA4 knockout mice in which the levels of retinal labels are uniformly reduced. These maps can be explained by either the saturation of EphA receptor mapping leading to the relative signaling model or by the reverse signaling conveyed by ephrin-As expressed by retinal axons.

**Conclusion:**

According to our model, experiments in Isl2/EphA3 knock-in mice test the interactions between effects of molecular labels and correlated activity during the development of neural connectivity. Correlated activity can partially restore topographic order even when molecular labels carry conflicting information.

## Background

In developing brain, connectivity is established under the influence of several factors. Neurons initially find appropriate targets based on the sets of chemical labels [1-3]. This *chemospecificity hypothesis *originally postulated by Roger Sperry [3] motivated the search for molecules that could be used as such cues and suggested the principles which direct growing axons to their targets. The precision of axonal projections is further fine-tuned through mechanisms based on correlated neural activity. These activity-dependent mechanisms are thought to implement the rules for modification of neuronal connections that were proposed by Donald Hebb [4]. Hebbian rules provide a paradigm through which sensory experience may influence the formation of the neuronal connectivity. This is in contrast to the molecular labels that are controlled primarily by genes. One of the central questions in the studies of the developing nervous system is how the influences of molecular labels are combined with Hebbian learning rules to yield connectivity that is both precise and adaptive [5].

The interaction between molecular cues and activity-dependent factors has been extensively studied on the example of the topographic projection from retina to superior colliculus (SC) [5]. Axons of retinal ganglion cells (RGC) form an orderly representation of the visual world in the brain, called topographic or retinotopic map [6]. This implies that two RGC axons, which originate from neighboring points in retina, terminate next to each other in the target region. Topographic maps are important to the organism, because they facilitate visual processing, which involves wiring local to the termination zone [7,8].

**Figure 1:**
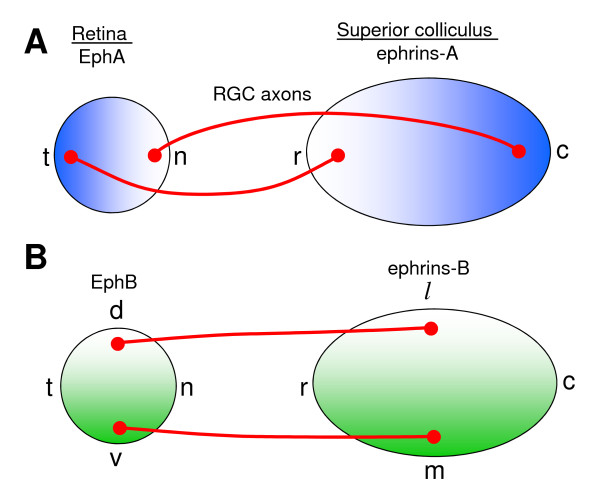
**Topographic map from retina to SC is dependent on two systems of reciprocal gradients**. (**A**) Temporal-nasal (TN) axis of the retina is mapped onto the anterior-posterior (AP) [also called rostral-caudal (RC)] axis of SC. This mapping is established through the graded expression of EphA receptors in retina and reciprocal expression of ligands from ephrin-A family in SC. (**B**) Similar principle applies to the mapping of dorsal-ventral (DV) axis of the retina onto medial-lateral (ML) axis of the target, where topography is formed based on gradients of EphB/ephrin-B pair1.

In case of retinotopic mapping, the role of molecular labels is played by the Eph family of receptor tyrosine kinases and their ligands ephrins [9-16]. The coordinate system is encoded in the retina through graded expression of Eph receptors by RGCs [17-24]. The recipient coordinates in SC are established by the graded expression of ephrin ligands [13,23,25,26]. The layout of the map in Figure 1 implies that axons expressing high level of EphA receptor are repelled by areas in the target expressing high level of ephrin-A. Such a repulsive cell signaling is triggered by ligand-receptor interaction through the activation of intracellular mechanisms with the net effect of preferred axonal extension towards the regions with low level of the ligand. Similarly it follows that the axons with high concentration of EphB label are attracted to the ephrin-B-rich regions (see however [27,28] for a discussion of possible alternatives). It is also possible that other molecules are involved in the formation of this projection. Thus, RGCs also express ephrins while Eph receptors are expressed by the cells in SC [16,29-31]. Additionally, it was demonstrated that BDNF, RGM, engrailed2 and Wnt3 play a role in the formation of retinocollicular projections [30-35].

The precision of topographic projections is further enhanced through mechanisms based on correlated neural activity [36]. Due to the presence of retinal waves during critical period of topographic map development, electric activity is similar in RGC axons neighboring in retina [37,38]. Correlated activity therefore provides additional information about axonal geometric origin in retina. Topographic maps are disrupted by blockade of the afferent activity with TTX [39,40], block of NMDA receptor in the target [41,42], or disruption of retinal waves during development [37,38,43,44]. The rough resolution of topographic maps is however preserved after these manipulations [37,42] due to remaining chemical labels. Therefore, it is assumed that patterned electric activity contributes to the refinement of topographic projection while Ephs and ephrins determine the global ordering of projections [45].

Insights into the mechanisms of map formation can be obtained from the experiments with mutant mice in which the distribution of chemical cues is altered. Thus, in Isl2/EphA3 knock-in mice a randomly chosen subset of retinal cells expresses additional member of EphA family of receptors, EphA3, that is not found in the RGCs of wild-type retina [46]. As a result, the connectivity between retina and SC changes. Simple models based on axonal sorting on the basis of the overall level of EphA expression succeed in explaining most of the phenotypes observed in Isl2/EphA3 mutants [47,48]. In some cases however the connectivity fails to change in these animals despite substantial modification of chemical labels [46]. Here we argue that the robustness of connectivity with respect to genetic manipulations may stem from the correlated activity-based Hebbian rules that remain operational in mutant retina. We suggest therefore that experience-dependent contribution arising from Hebbian rules may negate the effects of chemical labels. Our study suggests that experiments in Isl2/EphA3 mutant mice directly test the interplay between effects of molecular labels and correlated neural activity. The preliminary report of our findings can be found in Ref. [49].

## Results

### Topographic connectivity in wild type animals

The mechanisms for the formation of topographic maps have received an extensive attention from theorists [45,48,50-52]. One of the simplest models that is based on molecular labels and competition was first formulated in the prominent work of Prestige and Willshaw [53]. We developed a version of this model that involves signaling by known molecular labels, such as Eph receptors and ephrins, competition between axons for space or limiting factors in the target, and patterned electric activity [45,47]. The model includes repulsive cell signaling between the axons expressing EphA receptors and the dendrites in SC that express ephrin-A ligands. Our model postulates that the retino-collicular connectivity attempts to minimize the total number of EphA receptors bound to ligands evaluated for the entire system of axons. This view is consistent with the systems-matching ideas derived from the early lesion and transplantation experiments [54,55] and with Prestige and Willshaw mechanisms of competition [53]. The total number of bound/activated receptors is calculated using a form of mass action law (see Methods for more detail). It is then assumed that during retino-collicular development the connectivity evolves to minimize the total number of bound receptors through the iterative process of axon repositioning.

If the interactions in EphA/ephrin-A receptor/ligand pair were the only factor, all axons would project to the locations in the target with the lowest level of repellent (rostral in Figure 1). To prevent this, it is assumed that axons compete for space or limiting factors in the target [53]. Competition forces the axons with low levels of receptor to terminate in the areas with higher levels of repellent, since these axons are more indifferent to the ligand. Thus axonal competition in combination with EphA/ephrin-A repulsive signaling leads to the formation of the ordered topographic projections along RC axis. The importance of competition in the development of topographic connectivity was first emphasized by Prestige and Willshaw [53]. These authors also predicted the accurate distribution of labels that convey repulsive signals to axons, which was later confirmed with the experimental discovery of Ephs and ephrins [11]. Since then, competition has emerged as an important factor in many genetic and surgical experiments [13,56]. Similarly, it is thought that competition and chemoattraction in EphB/ephrin-B pair leads to the topographic mapping along ML axis if the entire system of axons tends to maximize the total number of EphB receptor bound to the ligand.

Our approach can also account for the effects of correlated activity on the topographic connectivity. These effects are governed by the Hebbian plasticity rules and can be included in the model using similar approach as with the molecular labels. When accounting for molecular labels, we suggested that topographic connectivity minimizes the total number of receptors bound/activated by ligands. This number is described in our model by the quantity called *E_chem_*. The effects of molecular labels in our model are accounted for by minimizing the value of *E_chem_*. Similarly the effects of correlated activity are included by minimizing another function called *E_act_*. Two functions *E_chem _*and *E_act _*are combined in our model additively

(1) $$E=E_{chem}+E_{act}$$

Because both *E_chem _*and *E_act _*are functions of retinocollicular connectivity, the minimum of their sum yields a set of projections that forms a compromise between molecular labels and the effects of correlated activity. To describe retinocollicular connectivity we minimize the sum described by equation (1) computationally or analytically (using pencil and paper, see Methods, section titled *Position of bifurcation*). The minimization approach postulated in equation (1) was pioneered by Fraser and Perkel before Eph receptors and ephrin ligands were known as the topographic labels [57]. We adopted this approach to describe Eph/ephrin-based signaling [47]. We also argued that additive form of interaction between molecular labels and the effects of correlated activity postulated in equation (1) is consistent with the experiments in ephrin-A knockout mice [45].

**Figure 2:**
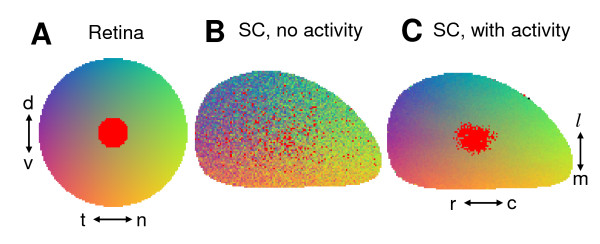
**Hebbian rules lead to an effective attraction in the target between axons with correlated activity**. (A) The origin of axons in the retina is color-coded. A small subset of axons in the area representing the middle of the retina is selected for tracing (red). (B) In the absence of correlated activity *E_act _*= 0 the topographic projection is imprecise and the set of axons selected in (A) forms a diffuse cloud in the target. (C) When *E_act _*≠ 0 the axons neighboring in retina (red) carry correlated activity they are attracted to each other. This effective attraction leads to the condensation of the axons into an almost precise image of the circle in the retina shown in (A). Thus Hebbian contribution *E_act _*leads to the sharpening of topographic projection.

For our subsequent discussion it is important to understand the effect of correlated activity-dependent contribution *E_act _*on topographic projection. This contribution was derived from the Hebbian learning rules [45] and has a simple intuitive meaning. We argue that Hebbian rules lead to an effective pair-wise attraction in the target between axons with correlated activity. This attraction makes topographic projection more precise (Figure 2). Sharpening of topographic projection due to the effects of correlated activity is consistent with experimental data on partial or full activity blockade [37].

In wild-type animals chemospecificity in the form of Eph/ephrin-based signaling and the Hebbian rules operate in unison leading to essentially the same ordering of axons. Mathematically this implies that minimum of *E_chem _*coincides with that of *E_act_*. The goal of both of these factors is to cooperate in making the topographic projection as sharp as possible. Below we will consider the connectivity in Isl2/EphA3 mutant mice for which the cooperation between chemical factors and Hebbian rules is disrupted.

### Retinocollicular connectivity in Isl2/EphA3 knock-in mice

**Figure 3:**
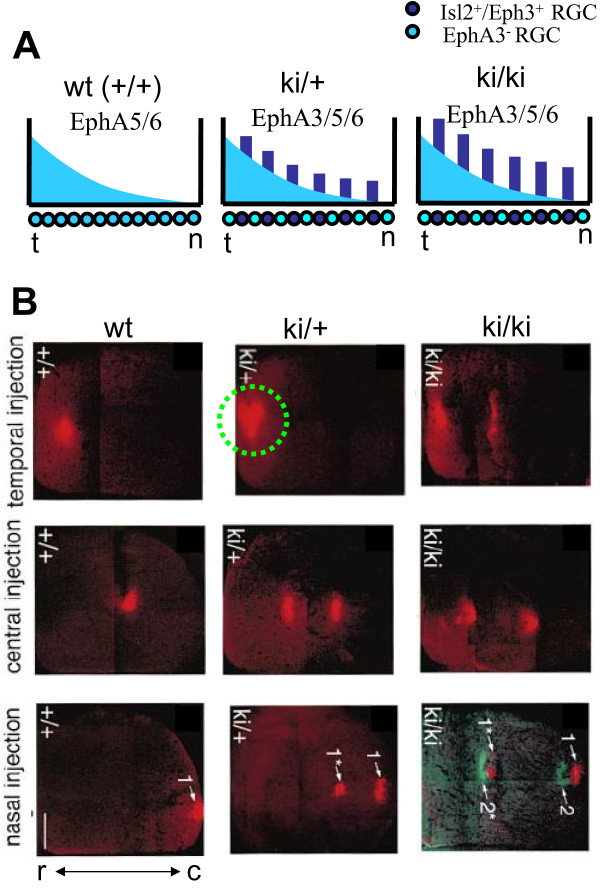
**Topographic map in Isl2/EphA3 knockin mice**. (A) Distribution of the EphA receptors in the retina of wild-type (left), heterozygous (center), and homozygous (right) knockins. (B) Results of anterograde tracing of axons after temporal, central, and nasal injections for these three animals adopted with slight modifications from [46]. In homozygous knockins (ki/ki, right column) the map is doubled for all three retinal locations. In heterozygous knockins (ki/+, central column) the map is doubled after nasal and central injection, but is single-valued after temporal injection (dashed circle).

The distribution of EphA receptors is altered in retinas of Isl2/EphA3 knock-in mice [46-48,51]. In these animals roughly 50% of retinal cells express additional receptor EphA3, which is not expressed by the wild-type cells. Thus, at each position in the retina there are two classes of cells: EphA3 positive and negative (EphA3+ and EphA3-, Figure 3A). This is accomplished by coexpression of ectopic EphA3 with LIM homeobox transcription factor Islet2 (Isl2) that is found in mosaic binary pattern of expression throughout RGC layer. EphA3+ axons experience a stronger repulsion from ligand in the target (SC) than EphA3- axons. Therefore EphA3+ and EphA3- axons neighboring in retina should terminate at different positions in the target. In particular the EphA3+ cells are expected to terminate at positions with lower level of repellent (Figure 1), i.e. at more rostral positions than EphA3- axons. This consideration leads to the prediction that tracing of axons projecting from a single locus in retina should yield two termination zones (TZs) in SC. One TZ (rostral) corresponds to EphA3+ axons, while the caudal TZ corresponds to EphA3- axons. This is indeed observed experimentally in homozygous EphA3 knock-ins [46] (Figure 3B).

Maps in Isl2/EphA3 homo- and heterozygote mutants are quantitatively different. The amount of additional EphA3 receptor is smaller in heterorozygous than in homozygous knockins by about a factor of two [46] (Figure 3A). This fact is reflected in a smaller separation between termination zones of the two types of axons (EphA3+ and -) in heterozygous knockins (Figure 3B, central column) compared to the homozygous case (Figure 3B, right column). When *temporal *axons are traced in Isl2/EphA3 heterozygotes however, two populations of axons blend completely forming a single termination zone (green circle in Figure 3). Thus, although chemical labels favor separation between two classes of axons having differing levels of EphA receptor, some additional factors compete with the effects of chemical labels. These additional factors restore the topographic quality of the projection from temporal retina despite the disruption of chemical labels. Here we investigated computationally what additional factors that could lead to the merging of two groups of axons (EphA3+ and -) in temporal retinas of heterozygous knockins.

### Correlated activity can mediate collapse of two maps in Isl2/EphA3 heterozygous knockins

Although EphA+ and EphA- axons carry different chemical labels that favor their separation in the target, some additional information about the axon retinal origin is still available. This information is represented in the electric activity of these axons that is correlated between cells neighboring in retina. Correlated neural activity could potentially restore the topographic order in temporal retina resulting in a single-valued map.

**Figure 4:**
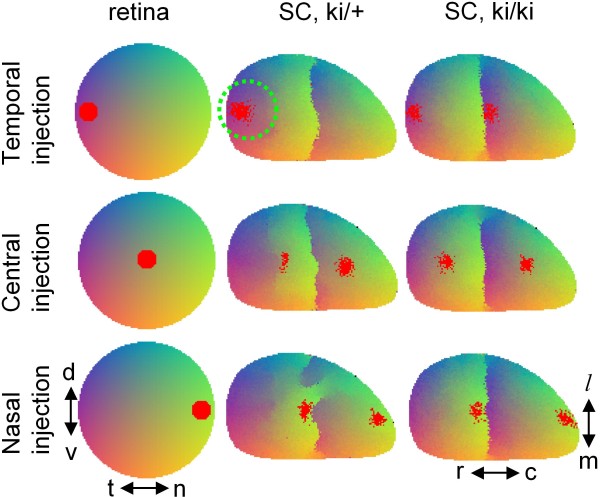
**The mapping in knock-ins obtained by the computational model**. The complete map structure is color-coded according to the axonal origin in retina as shown by the left column. In addition, the termination zones for a small subset of axons indicated by the red points are shown for temporal (upper row), central (center row) and nasal (lower row) labelings. This is to model the results of anterograde labeling as in Figure 3. The heterozygous knock-ins (central column) have smaller levels of the additional EphA receptor than homozygotes (right column). Comparison with experimental results in Figure 3 shows similarity. The maps in the central and right columns show the results of 6 simulations with different random initial connectivities. These panels show variability in projections, especially obvious in the heterozygous case (central column). The variability is mostly confined to the interface between wt and ki branches of the map, with the occasional formation of small folds (bottom panel).

Correlated activity enters our model through Hebbian learning rules leading to an effective attraction between axons with correlated activity in the target. Thus, the two classes of axons with different levels of chemical labels (EphA3+ and -) are attracted to each other by the Hebbian mechanisms because they originate from similar locations in the retina, which creates a potential for their collapse into a single TZ. When the difference in the levels of chemical labels is reduced, such as in the heterozygous knock-ins (Figure 4 central column), the activity-dependent factors restore topographic order.

**Figure 5:**
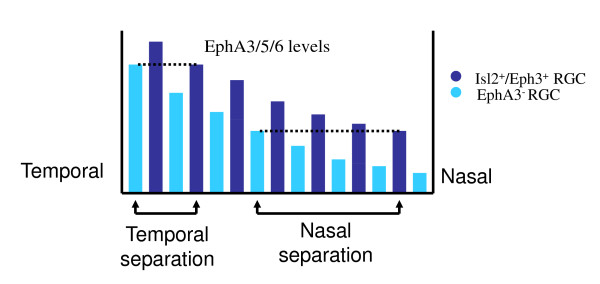
**The total amount of EphA receptor on the RGCs across the retina is composed of EphA3, EphA5 and EphA6 (EphA3/5/6)**. The separation between two sets of axons [EphA3+ (blue) and EphA3- (cyan)] is smaller if they originate from temporal retina. Because the gradient of the endogenous EphA receptor is maximal in the temporal retina EphA3- axons in this region have the smallest distance to the EphA3+ axons with similar overall levels of the receptor (dashed lines).

The effects of chemical labels are the weakest in temporal retina (Figure 5) which leads to the bifurcation in temporal rather than in the nasal retina. This is because the gradient of endogenous EphA is maximal in temporal retina [13] which leads to the weakest effect of exogenous EphA3 there [47]. The attraction between EphA3+ and EphA3- axons has the best opportunity to overcome the chemical factors in the temporal retina [see also discussion following equation (23) below]. Our model therefore reproduces the collapse of the two branches of topographic map into a single termination zone observed in heterozygous Isl2/EphA3 knock-in mice.

### Difference between transitions driven by correlated activity and noise

We argued previously [47] that collapse of two branches of the map in temporal retina of heterozygous Isl2/EphA3 knock-in mice may be caused by stochasticity and noise that limits map's precision. We have shown that if the separation between average locations of EphA3+ and EphA3- axons is smaller than the precision of the map, two types of projections may collapse into one. The finite precision of projections may be due to noise introduced by the stochastic nature of axon and dendrite branching [58]. In the present study we argued that an additional factor leading to the collapse of two branches of the map is the effective attraction due to Hebbian mechanisms. Below we analyze the differences between two types of map collapse, driven by noise and by correlated activity.

**Figure 6:**
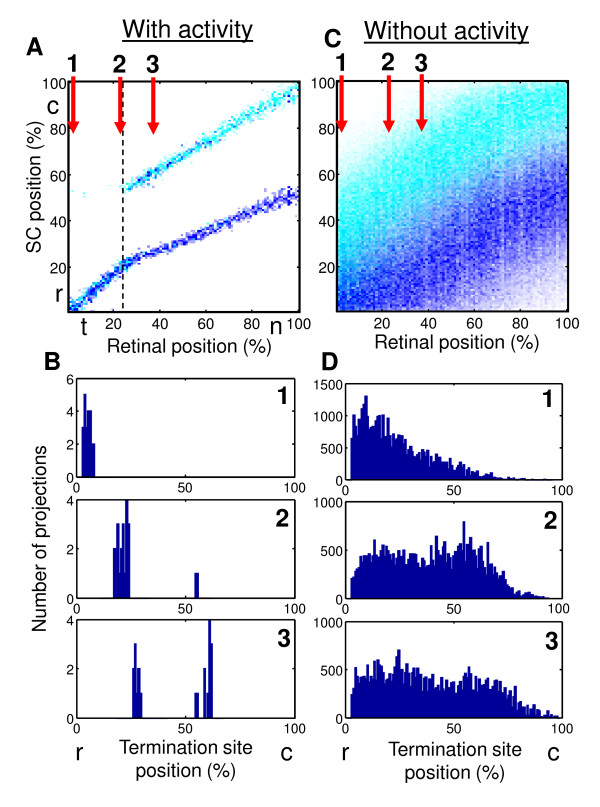
**Activity-driven collapse (A and B) of two types of projections (EphA3+ and EphA3-) is different from noise-driven collapse (C and D) (*E_act _*= 0)**. (A and C) The axonal positions in the target are shown as a function of the position of their origin in retina. Individual EphA3+/EphA3- axons are represented by blue/cyan points. (B and D) The distribution of terminal positions in the target for subset of axons originating from different retinal locations marked 1-3 in A and C. (B) includes projections for a single instance of mapping. (D) includes sum of projections for 1500 maps, which is necessary due to the degree of noise in the absence of correlated activity.

If collapse between two maps (EphA3+ and EphA3-) is driven by correlated activity (Figure 6A and 6B), the transition between doubled and single-valued maps is observed to be discontinuous. This implies that as the point of transition (Figure 6A point 2) is approached from the nasal direction one of the sets of axons (EphA3- i.e. WT) disappears at one location in the target and moves a finite distance to a different location to join another set of axons (EphA3+). In the noise-driven case (Figure 6C and 6D) the collapse is continuous and consists in blending of two broad distributions corresponding to EphA3+ and EphA3- axons as temporal regions of the retina are approached (Figure 6D, 1-3). As a result two distributions of axons do not truly blend in the noise-driven case. Indeed the EphA3- axons (cyan) in Figure 6C are always above EphA3+ axons (blue). This is in contrast to the correlated activity-driven case (Figure 6A), in which these two types of axons are truly mixed in the target if they originate from temporal retina. These features suggest that the transition that is driven by correlated activity is qualitatively different from the noise-driven collapse [47] in a way that can be distinguished experimentally.

### The saturation in Eph receptor signaling can mediate relative signaling mechanism suggested for Isl2/EphA3 ki/+ EphA4- mutants

The retinocollicular projection was also studied in mice in which the anomalous distribution of EphA3 receptor is combined with the decrease in the expression of EphA4 [48], i.e. Isl2/EphA3 ki/+ EphA4- mutants. EphA4 is normally expressed at a constant level throughout RGC layer. Topographic projections in EphA4 knockout mice are similar qualitatively to the Isl2/EphA3 case, i.e. maps are double-valued in homozygous and bifurcate in heterozygous knock-ins. The point of bifurcation however is shifted rostrally (temporally in the retinal coordinates) in these mutants. This implies that a smaller region of the map is occupied by single-valued projection. The doubled area of the map is extended in EphA4 mutants. Reber et al. [48] suggest that this shift is due to the relative signaling of EphA3 receptor. Indeed, when the overall level of receptor in retina is decreased, as in EphA4- mutants, the relative effect of adding EphA3 is stronger, thus leading to a larger area of doubled projection. This factor leads to an expansion of doubled map in hererozygous Isl2/EphA3 knockins, which explains why the bifurcation point is shifted rostrally.

To implement relative signaling by EphA receptors in our model we included a more realistic description for EphA receptors binding by the ephrin-A ligands. For example, the amount of bound receptor molecules obviously cannot exceed the number of ligand molecules present in the substrate. Similarly, the number of bound receptor molecules is limited by the total number of these molecules available in an axon. Consequently, when the number of bound EphA receptor molecules approaches the number of available molecules, the number of bound EphAs begins to saturate. Due to this saturation, the effect of additional EphA3 receptor is different for small/large overall EphA expressions levels. Axons with a lot of receptors (temporal) are less sensitive to the additional EphA3 receptor than the axons with the lower level of receptor (nasal). The saturation of EphA/ephrin-A signaling implements therefore the mechanism of relative signaling. Quantitatively saturation of EphA/ephrin-A signaling can be described by the simple mass action law as it is shown in Methods section [equation (3)].

**Figure 7:**
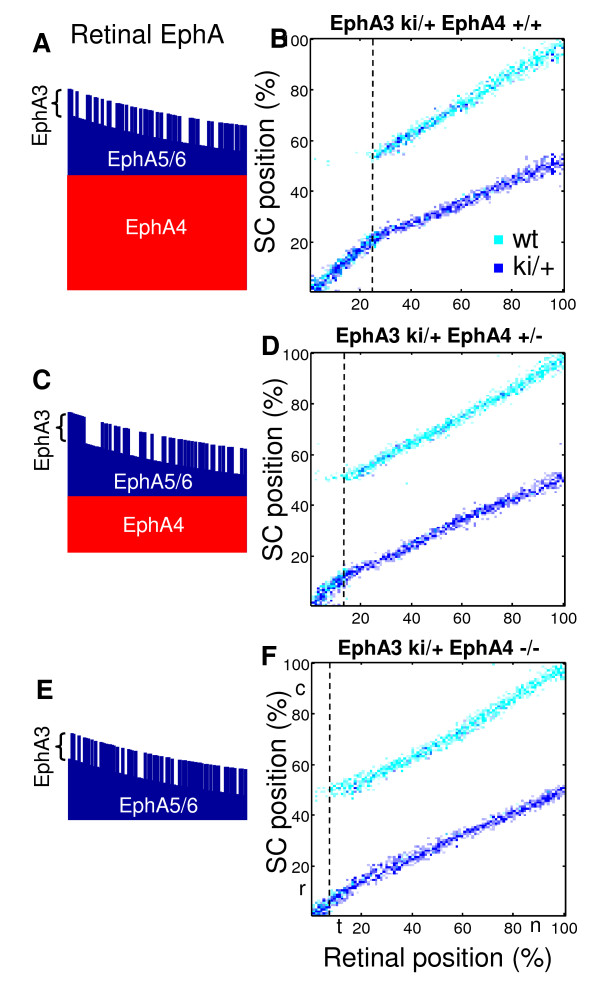
**Topographic maps in heterozygous EphA3 ki/+ knockins in the conditions of reduced overall levels of EphA receptor in the retina**. As the levels of receptor expression are reduced progressively from (A and B) EphA4+/+, to (C and D) EphA4+/-, and (E and F) EphA4-/-, the position of bifurcation (dashed line) shifts in the temporal direction in our simulations. The reason for this behavior is the desaturation of EphA receptor signaling leading to the increased impact of additional EphA3 receptor in EphA4 mutants. The levels of receptor are shown on the left in each case, with different forms of receptor indicated.

Saturation of EphA3 receptor signaling has implications for mapping in mutants in which the overall levels of receptor in retina are reduced, such as EphA4 knockout mice [48]. Indeed, because the degree of saturation of EphAs in these animals is lower throughout retina, the additional level of EphA3 should produce a bigger impact. This argument suggests that in the saturation model, as in the form of a relative signaling model, the doubled area of the map expands when the overall levels of receptor are reduced, i.e. in EphA4 knockout mutants. Consistently with this, when we simulate the maps in EphA3 ki/+ mutants, the position of bifurcation shifts in the temporal direction with decreasing levels of EphA4 present in the retina. This progression, corresponding to EphA4 +/+, EphA4 +/-, and EphA4 -/- mutants, in shown in Figure 7.

Saturation mechanism presented here implements relative signaling in the wide range of receptor densities below the saturation values. Thus, in our simulations presented in Figure 7 the maximum level of the receptor does not exceed one half of the saturation concentration, given by the dissociation constant for EphA/ephrin-A binding [see Methods for details].

### The effect of reverse ephrin-A signaling on the collapse of two maps

**Figure 8:**
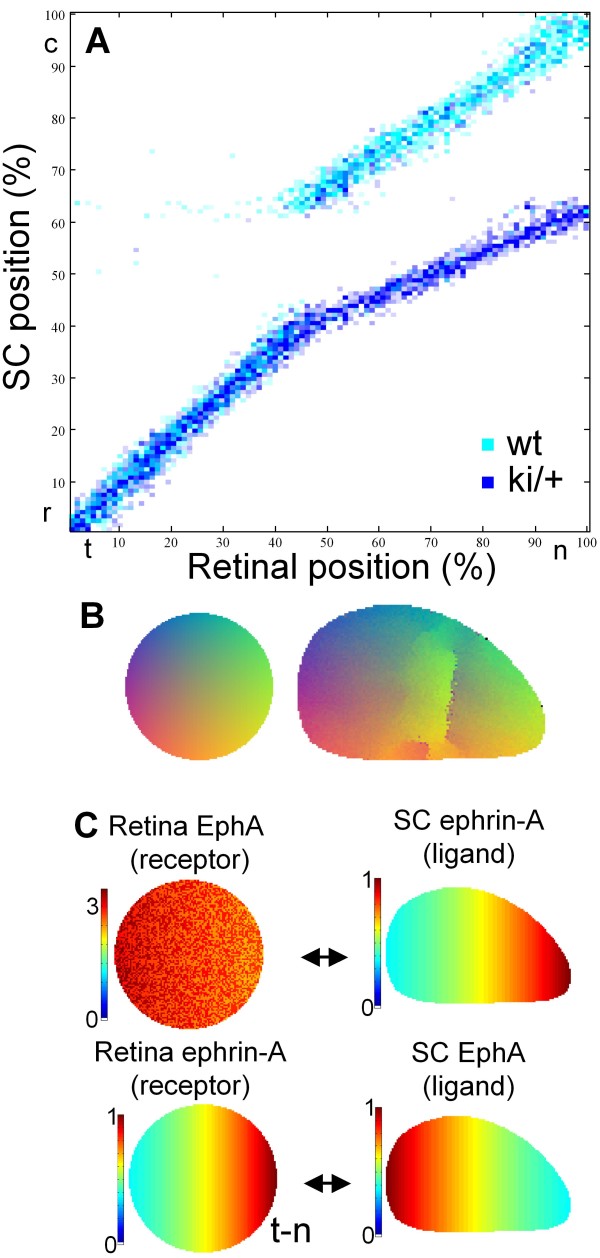
**The effect of reverse signaling on mapping in heterozygous EphA3 ki/+ mutants**. The bifurcation point shifts in the nasal direction when reverse signaling is turned on, expanding the single-valued region in the map. (A) t-n mapping, (B) map layout, and (C) distribution of molecular labels. The interactions between labels are indicated by the arrows.

EphA/ephrin-A receptor/ligand pair is thought to be able to implement the reverse signaling. Thus, retinal axons express ephrin-A ligands in addition to receptors. Ephrin-A ligands expressed by axons may interact with collicular EphA receptors and convey positional information to axons through the use of co-receptors [52,59]. This mechanism is known as reverse signaling by ephrin-A that is suggested to play the role of receptor. Here we investigated the implications of reverse signaling for the mapping in EphA3ki/+ mice. We show that qualitatively, the inclusion of reverse signaling leads to the expansion of single-valued part of the map (Figure 8).

To obtain our results in this section, we assumed that reverse signaling, i.e. binding and activation of retinal ephrin-A ligands by collicular EphA receptors, is ten times weaker than the forward signaling, i.e. activation of EphA receptor. First, majority of existing experimental data can be explained based on models that do not involve reverse signaling [45,53,60]. Second, striped assays experiments are consistent with reverse signaling being weaker than forward signaling (see below). Finally, recent experiments in math5 -/- mice are consistent with the model in which forward signaling and competition are the primary factors determining the map formation (Pfeiffenberger, Triplett, Yamada, Stafford, Litke, Sher, Koulakov, and Feldheim: *Competition is a driving force in topographic mapping*, submitted). However, even with such a weak interaction due to reverse signaling the effect on the position of the bifurcation is large (Figure 8).This effect is in essence similar to that of correlated neural activity. Indeed, reverse signaling mediated by retinal ephrin-A receptor carries information about axonal retinal origin that is independent from retinal EphA receptor. Thus, reverse signaling should contribute to topographic alignment of two branches of map, EphA3+ and EphA3-, i.e. to the expansion of single-valued (topographic) region of the map. In agreement with this argument, inclusion of reverse signaling in our model leads to the expansion of single-valued region (Figure 8) compared to forward signaling alone (Figure 7A).

The expansion of topographic (single-valued) map due to reverse signaling has implications for the mapping in EphA4 -/- (or +/-) mice. Indeed, if this mechanism takes place, collicular EphA4 should play a role of ligand for retinal ephrin-A. Because EphA4 has a gradient in colliculus [61], in EphA4 knockouts, the reverse signaling is reduced. Because this effect is opposite to the one in Figure 8 where reverse signaling is turned on, EphA4 knockouts should display a reduced single-valued map area compared to the wild-type. Thus, reverse signaling provides an alternative explanation to the reduction in single valued branch of the map in EphA4 knockout mice. These two explanations for the shift of the collapse point of two maps in Isl2/EphA3 ki/+ heterozygotes, i.e. relative signaling and reverse signaling, can be disambiguated in experiments on conditional EphA4 knockouts, in which the levels of EphA4 are reduced in retina only.

In summary, we suggest that reverse signaling, in which collicular EphA plays the role of ligand, while retinal ephrin-A plays the role of receptor, can explain the experiments in Isl2/EphA3 ki/+ EphA4 -/- mice. Experimentally, in these animals the single-valued or topographic part of the map is smaller than in Isl2/EphA3 ki/+ EphA4 +/+ mice [48]. As we just argued, in the reverse signaling model, collicular EphA4 plays the role of additional topographic label. When this label is removed, as in Isl2/EphA3 ki/+ EphA4 -/- mice, the topographic part of the map (single-valued) is expected to shrink compared to pure in Isl2/EphA3 ki/+ mice, similarly to experimental observations [48]. We thus suggest that reverse signaling model may provide an alternative to the relative signaling explanation, which is based on collicular rather than retinal EphA4.

The effect of removal/reduction in collicular EphA4 within the reverse signaling mechanism is expected to be opposite to the so-called masking effect [13,45]. Indeed, masking by collicular EphA4 sharpens the gradient of collicular ephrin-A available for signaling [45]. Thus masking by collicular EphA4 is improving the signaling by ephrin-A. If the levels of EphA4 are reduced, the signaling by the collicular ephrin-A is expected to weaken, because the gradient of available (not masked) ephrin-A becomes more shallow. Because the gradient of ephrin-A makes the map bifurcate by counteracting the effects of correlated neural activity, as we describe above, the double-valued part of the map is expected to shrink upon the reduction in collicular EphA4 within masking model. Thus masking model predicts an expansion of single-valued part of the map and a contraction of the double-valued part of the map in EphA4 -/+ or -/- Isl2/EphA3 ki/+ mutants compared to EphA4 +/+ Isl2/EphA3 ki/+. This behavior is opposite to what is observed experimentally and to the relative and reverse signaling mechanisms discussed above.

### Experimental predictions

The following features of the projection in heterozygous Isl2/EphA3 mutants could be used to confirm that the collapse of EphA3+ and EphA3- branches of the map is indeed driven by Hebbian factors. First, transition is spatially discontinuous. This implies that one projection (EphA3-) disappears as the location of retinal injection is moving from the nasal to the temporal direction. At the same time the disappearing termination site maintains a finite separation from the other branch of the map. This is in contrast to another possibility in which the two branches of the map merge continuously as the injection point is shifted temporarily. Second, it is the wild-type axons (EphA3-) that are actually making the transition. As evident from Figure 6A, the EphA3+ axons (blue) are shifting their locations smoothly as injection point is moved nasally past the collapse point. On the other hand the wild-type axons (cyan) are jumping a finite distance to join the EphA3+ group. Third, the termination site corresponding to the wild-type axons that is located more caudally in Figure 6B is gradually losing its strength as the point of collapse is approached. These three features of the transition are quite specific to the activity-driven collapse of the two maps and could be used to distinguish it from the noise-driven collapse experimentally.

## Discussion

In this study we investigated the interplay between effects of correlated activity and chemospecificity in the development of neural connectivity. We studied the model in which mechanisms based on the chemospecificity and correlated activity can operate independently and thus the contributions due to these two factors are combined additively [equation (1)]. We have shown previously that this form of interaction is sufficient to explain mapping in ephrin-A knockout mice [45]. Because correlations in the neural activity could be modulated by external stimuli, our model can combine the effects of genes and environment in the formation of neural connectivity.

We studied the maps in Isl2/EphA3 knock-in mice. In homozygous Isl2/EphA3 knock-ins the topographic map is fully doubled. This implies that an anterograde injection into any location in retina leads to two termination sites marked in SC (Figure 3B, right column). This form of connectivity can be explained using simple sorting of axons in the target on the basis of their overall expression level of EphA receptors [47]. These findings are reproduced in our model (Figure 4 right column). An interesting feature is observed in topographic maps of Isl2/EphA3 heterozygous knock-ins. In these mice, chemical labels alone should yield doubled map at each retinal location similar to the homozygous case [47]. This is because if chemical labels lead to the sorting of axons on the basis of their receptor level, heterozygous maps should differ from homozygous only in the extent of separation between two branches of the map. What is observed however is that the map is doubled in nasal retina as predicted by chemical labels but is single valued in temporal retina (Figure 3B, central column). The topographic map therefore experiences bifurcation when going from temporal to nasal retina. In other words, two branches of the doubled map collapse into a single-valued map when the observation point is moved temporarily. Our study focuses on the collapse (or bifurcation) that is observed in the maps of heterozygous knock-ins.

We argue that this feature is important because it may occur due to the interplay between the effects of correlated neural activity (Hebb) and chemical labels (Sperry). Indeed the chemical labels, such as Eph receptors and ephrins, favor doubled map throughout the knock-in retina. At the same time correlated neural activity favors single-valued mapping. This is because correlated activity carries additional information about an axonal geometric origin in retina that is independent on chemical labels. This information leads to an effective attraction between axons with correlated activity that originate from neighboring points in retina. As a result, activity-dependent attraction may overcome the separation produced by chemical labels and two branches of the map coalesce in temporal retina (Figure 6A), where the effects of chemical labels are the smallest (Figure 5). The point of bifurcation (Figure 6A, dashed line) is established from the balance of costs due to chemical labels (Sperry) and correlated activity (Hebb), as elaborated in Methods. We suggest experimental tests that could validate this scenario. Our conclusion about the importance of correlated activity in this mouse is confirmed by the recent study [62], in which the alignment of cortico-collicular projection is found to be activity-dependent.

### Topographic maps in EphA4 knockouts

Our model allows to reproduce the findings in Isl2/EphA3 ki/+ EphA4 knockout mice. Experimentally, in these mice the position of bifurcation between the single- and double-valued regions of the map is shifted in the temporal direction. Therefore, the double-valued region of the map is broadened in these animals, suggesting a stronger impact of additional EphA3. This finding is attributed to the relative signaling by the EphA receptors [48]. Indeed, because EphA4 is expressed uniformly throughout retina, reduction in the level of EphA4 in the knockout mice leads to the overall decrease in the level of EphA in the retina. This factor increases the relative impact of EphA3 that is added in the knockins. Our model suggests two explanations of this phenomenon. The first explanation is based on the saturation of the EphA signaling that emerges in the mass-action equations for the amount of receptor bound by ligands [equation (3)]. Saturation in the EphA signaling implements the relative signaling mechanism suggested previously [48]. We show that reducing EphA4 levels may desaturate the signaling by EphA receptor. This means that binding and activation of EphA receptors becomes more linear and less saturated when the overall level EphA is uniformly reduced. The effect of additional EphA receptor, such as EphA3, becomes bigger in EphA4 knockouts (Figure 7). Second, we suggest that the reverse signaling by ephrins-A may lead to a qualitatively similar phenomenon in EphA4 knockouts. This is because EphA4 knockouts have a reduced gradient of collicular EphA4, which leads to a decreased reverse signaling and narrower topographic (single-valued) region in the map, as suggested by Figure 8.

The two possible explanations to the phenotype observed in EphA4-Isl2/EphA3 ki/+ mice can be disambiguated if conditional knockouts, lacking EphA4 in retina only, are obtained. Thus, the EphA saturation model (relative signaling) will yield no noticeable differences in these animals compared to constituent knockouts, while the reverse signaling model should lead to the temporal shifts of the bifurcation point.

In our model, neither relative nor reverse signaling is necessary for bifurcation in heterozygous knockins. Indeed, as we show in the Methods section, the bifurcation in these animals can occur in temporal retina even if no relative signaling is assumed. The experiments in these animals therefore demonstrate the potential impact of correlated neural activity rather than the relative signaling mechanism. The relative mechanism however is a likely explanation of the changes in the maps observed in EphA4 knockouts as suggested by [48].

### Alternative models for retinocollicular connectivity formation

The most prominent alternative models include the dual gradient model (DGM) [52,63] and the servomechanism model (SVM) [51,57,64]. The former assumes that axons are influenced by two gradients in the target. In the simplest form axons are guided by gradients of two repellents: one having maximum in the rostral while the second having maximum in the caudal SC. The correct location for a termination zone for an axon is established through balancing the repulsive effects produced by these gradients. The second class of models represented by SVM assumes that axons terminate at locations with particular level of ligand corresponding to the expression level of receptor on the axon. This model was suggested to explain the mapping along the vertical dorso-ventral axis in the retina. Both of these models postulate the matching principle whereby the temporal axons will have preference to the rostral SC and will be repelled from the caudal SC. Similarly nasal axons preferentially grow in the caudal SC while are repelled from rostral SC in both DGM and SVM mechanisms. We suggest that the latter prediction of the matching principle models is not consistent with the striped assays experiment, in which nasal axons, when exposed to alternating stripes extracted from caudal and rostral SC, do not show any preference in their extension [65,66]. In addition, both DGM and SVM require competition between axons *in vivo *to explain topographic map compression/expansion observed after tectal/retinal lesions [57].

The model that we employ in this study is consistent with the striped assay experiments. Indeed, nasal axons express low levels of EphA receptor and, therefore, are weakly responsive to the difference between rostral and caudal stripes as observed in the striped assay experiments [65]. Temporal axons on the other hand have high levels of EphA receptor and, therefore, are repelled by the caudal stripes. This is because the latter have high levels of expression of the ephrin-A repellents (Figure 1). Based on this observation, we adopted the model that includes a single repellent and competition [45] because it is both parsimonious and consistent with the striped assay experiments.

### Other models for bifurcation in heterozygote mutants

Honda [51] put forward a model which obtains the double-valued maps in homozygote knock-ins. However the bifurcation of the maps in heterozygous case is not reproduced. Other models [47,52,67] yield the results resembling those is Figure 6C. These models therefore suggest an explanation to map's doubling (bifurcation) observed in heterozygous knock-ins. In these studies EphA3+ and EphA3- projections, although blended, are not fully merged in temporal retina. This implies that there is a bias for EphA3+/EphA3- axons to terminate more rostrally/caudally despite proximity of these projections. Here we suggest a qualitatively different solution in which two types of projections form the same termination site with no noticeable bias (Figure 6A).

## Conclusions

According to our model, experiments in Isl2/EphA3 knock-in mice test the interactions between effects of molecular labels and correlated neural activity during the development of neural connectivity. In these animals correlated activity can partially restore topographic order when molecular labels carry conflicting information.

## Methods

### The mass-action contribution (Sperry)

Our model is designed to predict the locations of terminations of retinal axons. More exactly, our model traces the behavior of synapses formed by axons. The synapses are defined by the weight matrix *W_ij_*, where the index *j *describes the number of retinal axon, while the index *i *is the number of the dendrite with which given synapse is formed. The weight matrix therefore describes the strength of connection between the axon number *j *and the dendrite number *i*. For simplicity we assume that each axon can form a single synapse with a dendrite and each dendrite can form a single synapse with an axon. We then define the affinity potential that is a function of the weight matrix. The affinity potential is a sum of the chemoaffinity (Sperry) and correlated activity-dependent (Hebb) contributions, as postulated by equation (1). The affinity potential is similar to the one used by us before [45,47] with the expression levels of chemical labels modified to address experiments in Isl2/EphA3 mutants [46]. The Sperry contribution is

(2) $$E_{chem}=\sum_{\alpha \beta }M_{\alpha \beta }\sum_{ij}W_{ij}B(R_{i}^{\alpha },L_{j}^{\beta },K_{\alpha \beta })$$

Here indexes α and β describe the chemical labels (α = EphA or B, β = ephrin-A or -B). The sum $\sum_{ij}W_{ij}B(R_{i}^{\alpha },L_{j}^{\beta },K_{\alpha \beta })$ defines the total number of receptors bound by ligands for a given pair. It depends on receptor and ligand expression levels $R_{i}^{\alpha }$ and $L_{j}^{\beta }$ of axon number *i *and dendrite number *j *respectively. It also depends on the dissociation constant for the pair of molecules *K*_αβ_. The total number of bound receptor-ligand pairs *B*(*R*, *L*, *K*) is determined by the receptor occupancy that can be derived from the mass-action law

(3) $$B(R,L,K)=\frac{R+L+K-\sqrt{{\left(R+L+K\right)}^{2}-4RL}}{2}.$$

This function describes receptor saturation by the ligand and vice versa. For example, the number of bound receptors cannot exceed the total number of ligand molecules present. In agreement with this, *B *→ *L *when the level of receptor is very high, i.e. *R *→ ∞. Conversely, *B *→ *R *when *L *→ ∞. For small levels of receptor and ligand, much smaller than the saturation concentration *K*, the number of bound receptor-ligand pairs is determined by a simpler expression

(4) $$B(R,L,K)\approx \frac{RL}{K}$$

This expression has been used by us in the previous studies. In this study, we adopt this simpler expression for all receptor-ligand pairs with the single exception of retinal EphA receptor bound by collicular ephrin-A for which we use equation (3). This is to account for the saturation of EphA signaling that could explain the relative signaling in EphA3 knockin mice as suggested by [48]. For this case we use the value of saturation concentration *K *= 7. For the interactions between EphB and ephrin-B we assumed no saturation, i.e. *K *= ∞.

Matrix *M*_αβ _defines the effects of receptor/ligand binding on retinal axons. Thus if *M*_αβ _is positive the interaction of receptors and ligands of given types is repulsive. For negative *M*_αβ _, the interaction is attractive. The absolute value of *M*_αβ _determines the strength of the effect of receptor *α *activation by ligand *β *on axons.

In our model we assumed that the dissociation constants between different EphA receptors and different ephrin-A ligands are the same. This was an approximation that was used to simplify our model. The same approximation was adopted for EphB/ephrin-B receptors/ligands. Although some *in vitro *data indicates that dissociation constants may differ within a family [11], conclusive data on the strength of binding *in vivo *is missing. Similarly, we assumed that the effects of receptor binding *M*_αβ _are the same within each receptor/ligand family. Because of these approximations, the receptor/ligand concentration was combined into a single number for every family (A and B).

### Derivation of the mass-action expression (3)

For a receptor-ligand pair the following equations describe the chemical equilibrium between bound and unbound receptors:

(5) $$\begin{array}{l} \frac{d[RL]}{dt}\sim [R][L]-K[R]=0\\ {}[R]+[RL]=R\\ {}[L]+[RL]=L \end{array}$$

Here [*R*], [*L*] are the levels of free (unbound) receptor and ligand, and [*LR*] is the level of bound receptor. *R *and *L *are the total levels of receptor and ligand present in an axon or on the substrate. By solving the system of equations (5) for *B *= [*RL*] we obtain equation (3).

### The Hebbian contribution

We adopted the Hebbian contribution from the previous studies:

(6) $$E_{act}=-\frac{1}{2}\sum_{ijml}C_{ij}W_{mi}W_{lj}U_{ml}$$

Here *C_ij _*is the correlation in electric activity between axons number *i *and *j*. This function describes the strength of similarity between axons as a function of their location is retina. The arbor function *U_ml _*on the other hand describes the strength of Hebbian interaction as a function of dendrite position in the target. The affinity potential defined by equations (1) through (6) is minimized using the stochastic procedure defined below.

### Optimization procedure

RGCs are arranged in retina on a square array restricted within a circle of radius equal to 48. These cells establish connections with a matching in the number of recipient cells square array of collicular dendrites. The square array is restricted to the oval area shown in the figures. Each axon is constrained to make connections with one and only one collicular dendrite for simplicity. We therefore assume that *W_mi _*= 1 for the pair of cells *m *and *l *that are connected and 0 for unconnected cells. This assumption implements the competition constraint described in the text.

We begin from a random set of connections that reflects the broad initial distribution of axons and their synapses in the target [68]. To minimize the affinity potential (1) we use an iterative stochastic optimization procedure. On each step of the algorithm two cells are chosen randomly. The cells are not necessarily neighboring in the target or in the retina. We then calculate the potential change in the affinity potential for the modification of retinocollicular connectivity in which these two cells exchange their positions in the target. This change in potential is defined by Δ*E*. The modification of connectivity is then implemented with probability

(7) $$p_{\text{exchange}}=\frac{1}{1+\exp(4\Delta E)}$$

Thus, if the potential is decreased as the result of this modification (Δ*E *< 0) the probability to accept this attempt is more than 1/2, leading therefore to the bias towards minimizing the overall value of potential. This step is repeated 10^7 ^times. The number of iterations is chosen to ensure the algorithm's convergence for the wild type distribution of the molecular labels.

### Receptor and ligand distributions

The parameters of the model are as follows. The distributions of molecular labels are

(8) $$R_{\text{EphA}}=\exp(-x/N)+\Delta R+R_{4}$$

(9) $$L_{\text{ephrin-A}}=\exp([x'-N]/N)$$

(10) $$R_{\text{EphB}}=\exp(-y/N)$$

(11) $$L_{\text{ephrin-B}}=\exp(-y'/N)$$

Here the horizontal and vertical coordinates in retina are *x *and *y*, while the collicular coordinates are denoted by *x*' and *y*'. All coordinates vary between 1 and *N*. The additional level of expression of EphA3 receptor Δ*R *is equal to 0, 0.45, and 0.9 for wild-type, heterozygous, and homozygous cases respectively. 50% of axons were chosen randomly to express EphA3 in each case. The level of EphA4 receptor *R*_4 _was equal to 2, 1, and 0 in EphA4+/+, +/-, and -/- mice correspondingly.

To model reverse signaling (Figure 8) we used the distribution of labels indicated in Figure 8C. The reverse signaling strength was 1/10th of that for the forward signaling. This estimate for the reverse signaling strength was derived from recent experiments on p75 receptor (a co-receptor mediating ephrin-A-based reverse signal) mutant mice that displayed ~10% rostral shifts of the termination zones [59]. Because direct interaction between retinal p75 and collicular BDNF cannot be ruled out, 10% provides an upper bound for the reverse signaling effect.

The matrix of affinities *M*_αβ _in equation (2) is

(12) $$M_{\text{EphA,ephrin-A}}=-M_{\text{EphB,ephrin-B}}=30\cdot K$$

(13) $$M_{\text{EphA,ephrin-B}}=M_{\text{EphA,ephrin-B}}=0$$

Negative/positive values of the matrix of affinities describe chemoattraction/repulsion. The zero values imply that there is no direct interaction between the "A" and "B" families of receptors and ligands [11]. *K *is the dissociation concentration [equation (2)]. *K *= 7 and *K *= ∞ was used for EphA/ephrin-A and EphB/ephrin-B interactions respectively. We therefore excluded saturation from the latter interaction for simplicity.

The parameters in equation (6) are as follows

(14) $$C_{ij}=\exp\left(-\left|{\vec{r}}_{i}-{\vec{r}}_{j}\right|/a\right)$$

(15) $$U_{ml}=\gamma \exp\left({\left[{\vec{r}}_{m}-{\vec{r}}_{l}\right]}^{2}/2b^{2}\right)$$

where *a *= 0.11*N *is the range of correlations in the retina [37,45] while *b *= 0.03*N *and γ = 0.25 are the range and the strength of Hebbian attraction in SC [45].

### Position of bifurcation

Here we calculate the location of the bifurcation point from the balance between Hebbian and Sperry contribution to the affinity potential. We will assume a simple form of mass-action law without saturation that is given by equation (4). The bifurcation in the map is associated with the interface between the single-valued and doubled maps. In the doubled map the Sperry contribution to the affinity functional is minimized, while the Hebbian contribution is increased by

(16) $$\Delta E_{act}=\int_{0}^{x}nd\tilde{x}\int_{0}^{N}dyU_{H}$$

Here *n *is the density of neurons (*n *= 1 in our model), *x *is the location of the interface, *U_H _*~ γ*nb*^2 ^is the Hebbian potential per neuron. The Hebbian potential is estimated here up to the numerical factor which depends on the exact geometry of the problem. In the single-valued part of the map the Hebbian contribution has the minimum possible value while the Sperry contribution is increased. The total increase in the Sperry contribution is

(17) $$\Delta E_{chem}=\int_{x}^{N}nd\tilde{x}\int_{0}^{N}dyU_{Sp}.$$

Here *U_sp _*is the Sperry contribution per neuron. The total affinity is minimum if

(18) $$\frac{dE}{dx}=\frac{d\Delta E_{chem}}{dx}+\frac{d\Delta E_{act}}{dx}=0$$

Due to equations (16) and (17) this implies that

(19) $$U_{H}=U_{Sp}$$

To evaluate the increase in the Sperry contribution in the area occupied by the single-valued contribution we notice that it is equal to

(20) $$U_{Sp}=M_{\text{EphA,ephrin-A}}\cdot \nabla R\nabla L\cdot \Lambda^{2}$$

Here Λ is the shift of the axons in the single-valued map from the location minimizing Sperry contribution. This shift is therefore equal to the separation between two branches of the doubled map. The gradients of the wild-type levels of receptor and ligand are denoted by ∇*R *and ∇*L*. Because this correction to potential per neuron describes deviation from the minimum it is quadratic in Λ. The assumption under which (20) is true is that Λ <<*N *i.e. maps separation in the doubled map is smaller than the size of the map.

To find Λ we notice that for doubled map *R*(*x *+ Λ) = *R*(*x*) + Δ*R*. This implies that the wild-type EphA3- axons with the level of EphA expression *R*(*x *+ Λ) terminate at the same location as the knockin EphA3+ axons with the receptor levels of *R*(*x*) + Δ*R*. Thus the separation between two maps is

(21) $$\Lambda =\frac{\Delta R}{\nabla R}$$

Combining (19), (20), and (21) we obtain the equation for the location of the point of doubling *x*

(22) $$\frac{\nabla L(x)}{\nabla R(x)}=\frac{U_{H}}{M_{\text{EphA,ephrin-A}}\Delta R^{2}}$$

The single-valued are of the map is defined by the condition

(23) $$\frac{\nabla L(x)}{\nabla R(x)}<\frac{U_{H}}{M_{\text{EphA,ephrin-A}}\Delta R^{2}},$$

which implies that the Hebb contribution is large. The doubled map region is defined by the opposite to equation (23) condition. This confirms the qualitative understanding that we derived from Figure 5 that collapse should occur more readily at location where the gradient of ligand is small and gradient of wild-type level of receptor is large i.e. in temporal retina, as observed experimentally (Figure 3).

## Authors' contributions

Both authors contributed equally to this work. All authors have read and approved the final version of the paper.
